# 3-Chloro­meth­yl-2-hy­droxy­benzaldehyde

**DOI:** 10.1107/S1600536812038421

**Published:** 2012-09-15

**Authors:** Wei-Wei Fu

**Affiliations:** a Key Laboratory of Functional Organometallic Materials of General Colleges and Universities in Hunan Province, Department of Chemistry and Materials Science, Hengyang Normal University, Hengyang 421008, People’s Republic of China

## Abstract

In the title compound, C_8_H_7_ClO_2_, the hydroxyl and aldehyde groups are co-planar with the benzene ring [maximum deviation 0.018 (3) Å], and the Cl—C—C plane is almost perpendicular to the benzene ring [dihedral angle 83.7 (2)°]. An intra­molecular O—H⋯O hydrogen bond occurs between the hydroxyl and aldehyde groups.

## Related literature

For related structures, see: Zondervan *et al.* (1997 ▶); Tang *et al.* (2010 ▶). For the synthesis, see: Song & Liu (2004 ▶).
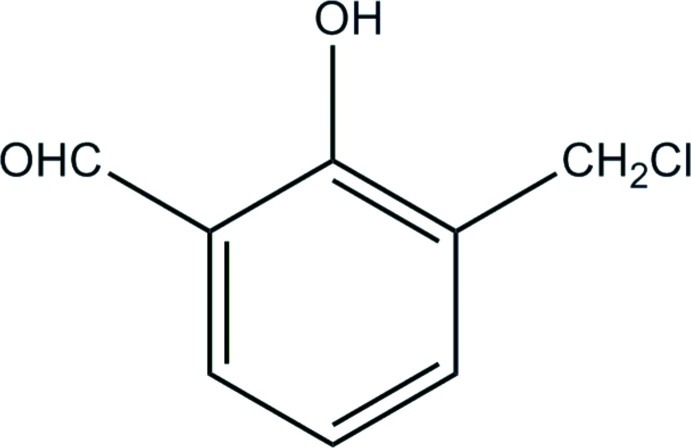


## Experimental

### 

#### Crystal data

C_8_H_7_ClO_2_
*M* *_r_* = 170.59
Orthorhombic, 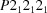

*a* = 4.483 (6) Å
*b* = 12.521 (18) Å
*c* = 13.71 (2) Å
*V* = 769.6 (19) Å^3^
*Z* = 4
Mo *K*α radiation
μ = 0.44 mm^−1^
*T* = 293 K
0.30 × 0.23 × 0.18 mm

#### Data collection

Bruker APEXII CCD diffractometer
Absorption correction: multi-scan (*SADABS*; Bruker, 2001 ▶) *T*_min_ = 0.88, *T*_max_ = 0.92
3730 measured reflections
1369 independent reflections
1075 reflections with *I* > 2σ(*I*)
*R*_int_ = 0.052

#### Refinement

*R*[*F* ^2^ > 2σ(*F* ^2^)] = 0.033
*wR*(*F* ^2^) = 0.133
*S* = 0.98
1369 reflections
101 parameters
H-atom parameters constrained
Δρ_max_ = 0.31 e Å^−3^
Δρ_min_ = −0.32 e Å^−3^
Absolute structure: Flack (1983 ▶), 6571 Friedel pairs
Flack parameter: −0.06 (13)

### 

Data collection: *APEX2* (Bruker, 2007 ▶); cell refinement: *SAINT* (Bruker, 2007 ▶); data reduction: *SAINT*; program(s) used to solve structure: *SHELXTL* (Sheldrick, 2008 ▶); program(s) used to refine structure: *SHELXTL*; molecular graphics: *SHELXTL*; software used to prepare material for publication: *SHELXTL*.

## Supplementary Material

Crystal structure: contains datablock(s) I, global. DOI: 10.1107/S1600536812038421/xu5608sup1.cif

Structure factors: contains datablock(s) I. DOI: 10.1107/S1600536812038421/xu5608Isup2.hkl

Additional supplementary materials:  crystallographic information; 3D view; checkCIF report

## Figures and Tables

**Table 1:** Hydrogen-bond geometry (Å, °)

*D*—H⋯*A*	*D*—H	H⋯*A*	*D*⋯*A*	*D*—H⋯*A*
O1—H1⋯O2	0.82	1.91	2.628 (5)	146

**Fig. 1.**
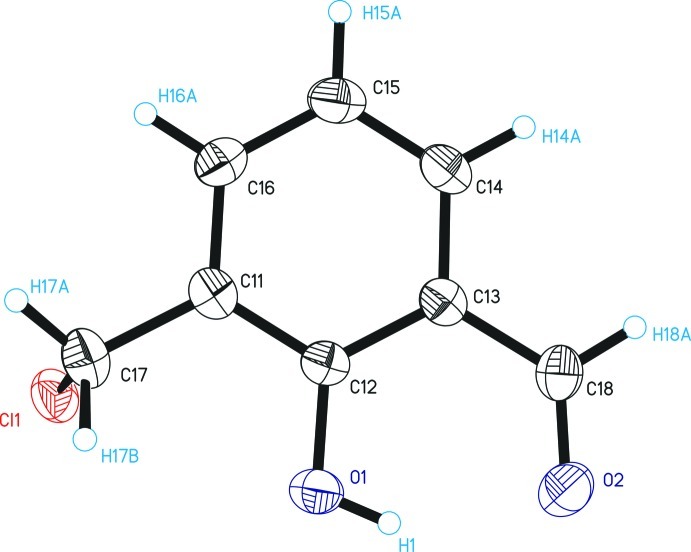
The molecular structure of title compound, with atom labels and 30% probability displacement ellipsoids for all atoms.

**Fig. 2.**
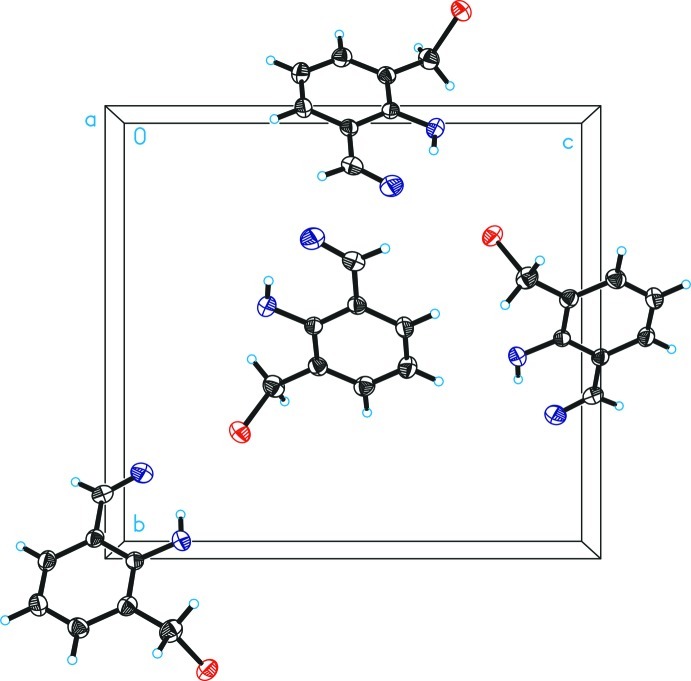
The cell packing diagram of title compound, viewed down the a axis.

## supplementary crystallographic information

### Comment

5-(Chloromethyl)-2-hydroxybenzaldehyde are well investigated just as it can be a precusor to an inhibitor-schiff bases for metal. However, just as we synthesize 5-(chloromethyl)-2-hydroxybenzaldehyde following one mehtod(Song & Liu, 2004) an unexpected byprodut 3-(chloromethyl)-2-hydroxybenzaldehyde was found and its cystal structure was determined.

### Experimental

Following a reference (Song *et al.* 2004), salicylaldehyde (30.5 g), paraformaldehyde (13.5 g) and conc. HCl (150 ml) were mixed and stirred at room temperature for 48 h. The precipitated benzylchloride derivatives which mostly are 5-(chloromethyl)-2-hydroxybenzaldehyde were filtered off then washed with 0.5% NaHCO_3_ solution and water slightly. These humid precipitate were then dried in vac. for about 3 months. There are block colorless crystals appeared on the surface of precipitate.

### Refinement

H atoms were positioned geometrically and refined using a riding model.

### Crystal data

**Table:**

C_8_H_7_ClO_2_	*F*(000) = 352
*M**_r_* = 170.59	*D*_x_ = 1.472 Mg m^−^^3^
Orthorhombic, *P*2_1_2_1_2_1_	Mo *K*α radiation, λ = 0.71073 Å
Hall symbol: P 2ac 2ab	Cell parameters from 1369 reflections
*a* = 4.483 (6) Å	θ = 2.2–25.0°
*b* = 12.521 (18) Å	µ = 0.44 mm^−^^1^
*c* = 13.71 (2) Å	*T* = 293 K
*V* = 769.6 (19) Å^3^	Block, colorless
*Z* = 4	0.30 × 0.23 × 0.18 mm

### Data collection

**Table:**

Bruker APEXII CCD diffractometer	1369 independent reflections
Radiation source: fine-focus sealed tube	1075 reflections with *I* > 2σ(*I*)
Graphite monochromator	*R*_int_ = 0.052
ω scan	θ_max_ = 25.2°, θ_min_ = 2.2°
Absorption correction: multi-scan (*SADABS*; Bruker, 2001)	*h* = −5→5
*T*_min_ = 0.88, *T*_max_ = 0.92	*k* = −15→14
3730 measured reflections	*l* = −16→16

### Refinement

**Table:**

Refinement on *F*^2^	Secondary atom site location: difference Fourier map
Least-squares matrix: full	Hydrogen site location: inferred from neighbouring sites
*R*[*F*^2^ > 2σ(*F*^2^)] = 0.033	H-atom parameters constrained
*wR*(*F*^2^) = 0.133	*w* = 1/[σ^2^(*F*_o_^2^) + (0.*P*)^2^] where *P* = (*F*_o_^2^ + 2*F*_c_^2^)/3
*S* = 0.98	(Δ/σ)_max_ < 0.001
1369 reflections	Δρ_max_ = 0.31 e Å^−^^3^
101 parameters	Δρ_min_ = −0.32 e Å^−^^3^
0 restraints	Absolute structure: Flack (1983), 6571 Friedel pairs
Primary atom site location: structure-invariant direct methods	Flack parameter: −0.06 (13)

### Special details

**Table:**

Geometry. All e.s.d.'s (except the e.s.d. in the dihedral angle between two l.s. planes) are estimated using the full covariance matrix. The cell e.s.d.'s are taken into account individually in the estimation of e.s.d.'s in distances, angles and torsion angles; correlations between e.s.d.'s in cell parameters are only used when they are defined by crystal symmetry. An approximate (isotropic) treatment of cell e.s.d.'s is used for estimating e.s.d.'s involving l.s. planes.
Refinement. Refinement of *F*^2^ against ALL reflections. The weighted *R*-factor *wR* and goodness of fit *S* are based on *F*^2^, conventional *R*-factors *R* are based on *F*, with *F* set to zero for negative *F*^2^. The threshold expression of *F*^2^ > σ(*F*^2^) is used only for calculating *R*-factors(gt) *etc*. and is not relevant to the choice of reflections for refinement. *R*-factors based on *F*^2^ are statistically about twice as large as those based on *F*, and *R*- factors based on ALL data will be even larger.

### Fractional atomic coordinates and isotropic or equivalent isotropic displacement parameters (Å^2^)

**Table:**

	*x*	*y*	*z*	*U*_iso_*/*U*_eq_	
Cl1	0.5383 (2)	0.21212 (5)	0.21927 (5)	0.0606 (3)	
O1	0.3758 (7)	−0.05428 (14)	0.16474 (16)	0.0558 (7)	
H1	0.2636	−0.1057	0.1598	0.084*	
O2	0.0222 (7)	−0.18989 (15)	0.07502 (19)	0.0640 (7)	
C11	0.6216 (8)	0.07288 (19)	0.0685 (2)	0.0440 (7)	
C12	0.4289 (7)	−0.01314 (17)	0.0756 (2)	0.0384 (7)	
C13	0.2937 (8)	−0.05626 (17)	−0.0076 (2)	0.0403 (7)	
C14	0.3593 (9)	−0.0101 (2)	−0.0985 (2)	0.0509 (9)	
H14A	0.2719	−0.0378	−0.1546	0.061*	
C15	0.5488 (10)	0.0745 (2)	−0.1057 (2)	0.0560 (9)	
H15A	0.5907	0.1043	−0.1663	0.067*	
C16	0.6779 (9)	0.1157 (2)	−0.0228 (3)	0.0507 (8)	
H16A	0.8062	0.1738	−0.0282	0.061*	
C17	0.7744 (9)	0.1173 (3)	0.1567 (3)	0.0572 (9)	
H17A	0.9581	0.1522	0.1371	0.069*	
H17B	0.8252	0.0594	0.2007	0.069*	
C18	0.0905 (9)	−0.14473 (19)	−0.0012 (2)	0.0504 (9)	
H18A	0.0050	−0.1695	−0.0587	0.060*	

### Atomic displacement parameters (Å^2^)

**Table:**

	*U*^11^	*U*^22^	*U*^33^	*U*^12^	*U*^13^	*U*^23^
Cl1	0.0771 (7)	0.0537 (4)	0.0510 (5)	−0.0025 (4)	−0.0076 (4)	−0.0126 (3)
O1	0.078 (2)	0.0496 (10)	0.0400 (12)	−0.0038 (11)	−0.0019 (12)	0.0056 (7)
O2	0.074 (2)	0.0533 (9)	0.0649 (15)	−0.0091 (11)	0.0058 (15)	0.0042 (9)
C11	0.045 (2)	0.0425 (11)	0.0443 (16)	0.0089 (12)	−0.0040 (13)	−0.0039 (10)
C12	0.0424 (18)	0.0371 (9)	0.0358 (13)	0.0089 (11)	0.0027 (14)	0.0003 (9)
C13	0.042 (2)	0.0386 (10)	0.0398 (16)	0.0085 (12)	−0.0002 (13)	−0.0032 (9)
C14	0.067 (3)	0.0507 (12)	0.0353 (15)	0.0082 (14)	−0.0039 (16)	−0.0031 (10)
C15	0.067 (3)	0.0583 (14)	0.0427 (17)	0.0024 (16)	0.0070 (17)	0.0048 (11)
C16	0.050 (2)	0.0474 (12)	0.055 (2)	−0.0035 (14)	0.0055 (17)	0.0036 (12)
C17	0.054 (2)	0.0597 (14)	0.058 (2)	0.0024 (15)	−0.0145 (17)	−0.0069 (14)
C18	0.053 (2)	0.0412 (11)	0.057 (2)	0.0004 (12)	0.0011 (17)	−0.0076 (11)

### Geometric parameters (Å, º)

**Table:**

Cl1—C17	1.808 (4)	C13—C18	1.437 (4)
O1—C12	1.348 (4)	C14—C15	1.362 (5)
O1—H1	0.8200	C14—H14A	0.9300
O2—C18	1.227 (4)	C15—C16	1.376 (5)
C11—C12	1.384 (4)	C15—H15A	0.9300
C11—C16	1.386 (5)	C16—H16A	0.9300
C11—C17	1.496 (5)	C17—H17A	0.9700
C12—C13	1.400 (4)	C17—H17B	0.9700
C13—C14	1.404 (4)	C18—H18A	0.9300
			
C12—O1—H1	109.5	C14—C15—H15A	120.2
C12—C11—C16	118.6 (3)	C16—C15—H15A	120.2
C12—C11—C17	121.2 (3)	C15—C16—C11	121.7 (3)
C16—C11—C17	120.2 (3)	C15—C16—H16A	119.2
O1—C12—C11	118.1 (3)	C11—C16—H16A	119.2
O1—C12—C13	121.0 (3)	C11—C17—Cl1	111.1 (3)
C11—C12—C13	120.9 (3)	C11—C17—H17A	109.4
C12—C13—C14	118.2 (3)	Cl1—C17—H17A	109.4
C12—C13—C18	121.5 (3)	C11—C17—H17B	109.4
C14—C13—C18	120.3 (3)	Cl1—C17—H17B	109.4
C15—C14—C13	121.0 (3)	H17A—C17—H17B	108.0
C15—C14—H14A	119.5	O2—C18—C13	124.5 (3)
C13—C14—H14A	119.5	O2—C18—H18A	117.8
C14—C15—C16	119.6 (3)	C13—C18—H18A	117.8
			
C16—C11—C12—O1	180.0 (3)	C18—C13—C14—C15	179.3 (3)
C17—C11—C12—O1	1.8 (4)	C13—C14—C15—C16	−0.1 (5)
C16—C11—C12—C13	0.1 (4)	C14—C15—C16—C11	0.4 (5)
C17—C11—C12—C13	−178.1 (3)	C12—C11—C16—C15	−0.4 (5)
O1—C12—C13—C14	−179.7 (3)	C17—C11—C16—C15	177.8 (3)
C11—C12—C13—C14	0.2 (4)	C12—C11—C17—Cl1	−84.7 (3)
O1—C12—C13—C18	0.9 (4)	C16—C11—C17—Cl1	97.2 (4)
C11—C12—C13—C18	−179.3 (3)	C12—C13—C18—O2	−0.9 (5)
C12—C13—C14—C15	−0.2 (5)	C14—C13—C18—O2	179.7 (4)

### Hydrogen-bond geometry (Å, º)

**Table:**

*D*—H···*A*	*D*—H	H···*A*	*D*···*A*	*D*—H···*A*
O1—H1···O2	0.82	1.91	2.628 (5)	146
